# Recalibrating the significance of the decline effect in fish ocean acidification research

**DOI:** 10.1371/journal.pbio.3002113

**Published:** 2023-05-09

**Authors:** Andrew J. Esbaugh

**Affiliations:** Department of Marine Science, University of Texas at Austin, Marine Science Institute, Port Aransas, Texas, United States of America

## Abstract

The recently described decline effect in ocean acidification impacts on fish behavior should not be equated with negligible effects. This Perspective article uses existing mechanistic data to argue for continued research and cautions against “throwing the baby out with the bathwater.”

## Teaser

The recently described decline effect in ocean acidification impacts on fish behaviour should not be equated with negligible effects. Here, existing mechanistic data are used to argue for continued research and cautions against “throwing the baby out with the bathwater.”

The past few years have seen a seismic shift in the scientific consensus of ocean acidification and its impacts on fishes, particularly the adverse effects on behaviour. Foundational early work on coral reef fishes detailed olfactory disturbances that left fish unable to detect or discriminate predator cues and necessary habitat settlement cues—both of which were held up as potentially serious consequences of ocean acidification that may threaten global fish populations [1]. A decade later, Clark and colleagues published a rigorous follow-up that questioned the reproducibility of the early work on fish behaviour [2], and while several design aspects were disputed [3], the prevailing opinions on the behavioural effects of ocean acidification on fishes began to change. The recently published meta-analysis by Clements and colleagues [4] reinforced this shift by demonstrating a decline in effect size response ratios over time in studies exploring the impacts of ocean acidification on fish behaviour. The authors argued that ocean acidification has negligible effects on fish behaviour. More alarming was the determination that a University investigative panel concluded that a prominent author of the early ocean acidification studies committed scientific misconduct in the form of data fabrication and falsification [5]. This has led to one retraction of a high-impact work on coral reef fishes, although as of this writing, no ocean acidification papers have been retracted nor any expressions of concern been issued. Nonetheless, guilt by association has coloured the field of ocean acidification and fish behaviour. Despite all of this, I would urge the scientific community to remember the classic idiom and not “throw the baby out with the bathwater”.

**Fig 1:**
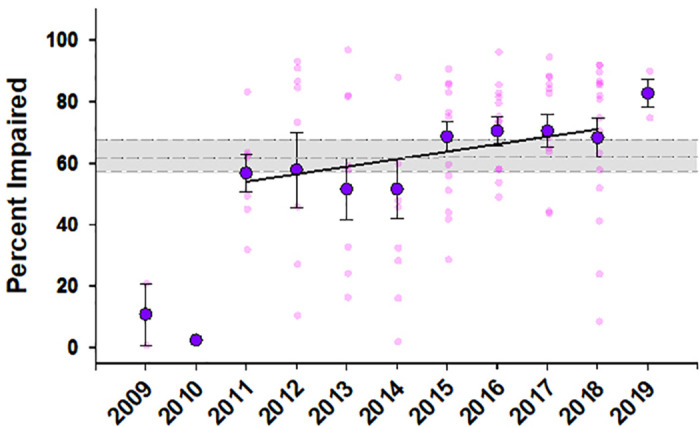
The mean study effect sizes reported by Clements and colleagues [4] represented as a measure of percent impairment. In this case, the data express effect sizes as a percent decline in behavioural response as a result of ocean acidification exposure, with control values set at 100%. The grey box denotes the mean and upper and lower 95% confidence intervals (62.6 ± 5.2%; *N* = 91). The pink dots denote individual study means, and the purple dots denote the mean ± SEM of a given year. The solid black line denotes the meta-regression line for 2011–2018 (*p* = 0.03; R^2^ = 0.05; *N* = 88). All data are publicly available through Dryad under doi: 10.5061/dryad.866t1g1vr.

Whether designated “extreme” or not—there has been continued discourse regarding the treatment of zero values and the resulting impact on the magnitude of the observed decline effect [6,7]—it is clear that the impacts of ocean acidification on fish behaviour meet the criteria of a decline effect. But the significance of the decline effect is in danger of being overinterpreted. The data of Clements and colleagues show a steep decline from a response ratio of approximately 4 to below 0.5 from 2009 to 2015, after which the response ratio stays relatively stable until 2019. But does this demonstrate that there are no effects of ocean acidification on fish behaviour as suggested by the authors? The average response ratio of the 91 studies in the Clements and colleagues’ database is 0.65 ± 0.09 (mean ± SEM), or 0.56 ± 0.06 if 2009 and 2010 are omitted (*N* = 88 studies). To provide a different context from Clements and colleagues, I have expressed each study effect size as a percent of control (i.e., control values are set to 100%, and ocean acidification values were calculated based on the reported effect sizes (Fig 1). In this context, the effects of ocean acidification in the post-decline effect data (i.e., post-2010) would still equate to an average 35% decrease in expected response. Note that a linear regression for effect values from 2011 to 2018 is provided, and, although visually, the slope suggests a continued decline effect over this time period the R^2^ of this relationship is only 0.05 (*p* = 0.03; *N* = 88; note that 2019 was omitted owing to the small number of studies). If a 35% decline in behavioural response was tied to survivorship it would surely garner scientific attention. This is not to say ocean acidification documented behavioural effects should be viewed as life or death—although some should—but instead to demonstrate that interpreting effect size requires context. The question is not whether there is literature support for ocean acidification effects on fish behaviour, but what species are affected and are the documented effects ecologically meaningful. These are questions that require careful consideration and research depending on the nature of the behaviour, the test design, the target species, and ecosystem. But I would argue that the unusually high effect sizes of the early behavioural work on fishes should not be the barometer for a meaningful effect of ocean acidification. The individual results of well-designed and thoroughly evaluated studies should not be overlooked because of documented resilient species and behaviours in the literature, or the alleged misconduct of another actor in the field.

It is also important to recognise that several demonstrated behavioural effects in fishes have strong underlying mechanistic support, of which I will provide 3 examples. The first relates to the contested olfactory responses of fishes following exposure to ocean acidification. Researchers used electrophysiological measurements from olfactory nerves of the European seabass to demonstrate that near-future CO_2_ levels impair olfactory sensitivity to a wide variety of amino acids, bile acids, and body fluids with the overall conclusion that fish must be 42% closer to an odour source for proper detection [8]. Similar electrophysiological insight has been used to validate impaired auditory performance thought to stem from ocean acidification induced otolith overgrowth. In this case, researchers demonstrated that larval snapper (*Chrysophrys auratus*) reared under ocean acidification conditions had significantly reduced low frequency hearing sensitivity, as detected via auditory evoked potentials [9]. A final example relates to the reversible and dose-dependent effects of ocean acidification on driving anxiety behaviours in fishes [10,11]. An elegant series of pharmacological and CO_2_ exposures demonstrated that a GABA_A_-receptor agonist (muscimol) exacerbated CO_2_-induced anxiety in CO_2_-exposed rockfish with no impact on control fish, while a GABA_A_-receptor antagonist (gabazine) drove anxiety in controls with no effects on CO_2_ exposed fish [11]. These findings were consistent with the hypothesis that ocean acidification–induced changes in acid–base chemistry were sufficient to reverse the flow of chloride ions through GABA_A_-receptors with corresponding changes in behaviour.

It is an understatement to say it is concerning that researchers were unable to reproduce the effects of ocean acidification on coral reef fish behaviour, particularly when confronted with the realities of scientific misconduct. The decade-old worries that ocean acidification may pose a serious and widespread threat to global fish populations is also, in hindsight, an overstatement, and current scientific discourse on the matter should reflect these changing views. While it is tempting to hypothesise that all fishes will react similarly to ocean acidification, the reality is that fishes are the most diverse vertebrate group on the planet and live in a wide range of habitats. It should not be surprising that sensitive and resilient species have been identified. But it remains important to acknowledge the robust work that supports behavioural impairments of ocean acidification in fishes, particularly where backed by thorough mechanistic physiology. If we are to fully understand the effects of climate change on fisheries health and food security, it seems reasonable that the effects of CO_2_ on fish behaviour continue to be explored by the scientific community.

## Dryad DOI

10.5061/dryad.866t1g1vr [12].
